# Low Gilbert Damping Constant in Perpendicularly Magnetized W/CoFeB/MgO Films with High Thermal Stability

**DOI:** 10.1038/s41598-018-31642-9

**Published:** 2018-09-06

**Authors:** Dustin M. Lattery, Delin Zhang, Jie Zhu, Xudong Hang, Jian-Ping Wang, Xiaojia Wang

**Affiliations:** 1 Department of Mechanical Engineering, University of Minnesota, Minneapolis, MN 55455 USA; 2 Department of Electrical and Computer Engineering, University of Minnesota, Minneapolis, MN 55455 USA

## Abstract

Perpendicular magnetic materials with low damping constant and high thermal stability have great potential for realizing high-density, non-volatile, and low-power consumption spintronic devices, which can sustain operation reliability for high processing temperatures. In this work, we study the Gilbert damping constant (*α*) of perpendicularly magnetized W/CoFeB/MgO films with a high perpendicular magnetic anisotropy (PMA) and superb thermal stability. The *α* of these PMA films annealed at different temperatures (*T*_ann_) is determined *via* an all-optical Time-Resolved Magneto-Optical Kerr Effect method. We find that *α* of these W/CoFeB/MgO PMA films decreases with increasing *T*_ann_, reaches a minimum of *α* = 0.015 at *T*_ann_ = 350 °C, and then increases to 0.020 after post-annealing at 400 °C. The minimum *α* observed at 350 °C is rationalized by two competing effects as *T*_ann_ becomes higher: the enhanced crystallization of CoFeB and dead-layer growth occurring at the two interfaces of the CoFeB layer. We further demonstrate that *α* of the 400 °C-annealed W/CoFeB/MgO film is comparable to that of a reference Ta/CoFeB/MgO PMA film annealed at 300 °C, justifying the enhanced thermal stability of the W-seeded CoFeB films.

## Introduction

Since the first demonstration of perpendicular magnetic tunnel junctions with perpendicular magnetic anisotropy (PMA) Ta/CoFeB/MgO stacks^1^, interfacial PMA materials have been extensively studied as promising candidates for ultra-high-density and low-power consumption spintronic devices, including spin-transfer-torque magnetic random access memory (STT-MRAM)^2,3^, electrical-field induced magnetization switching^4–6^, and spin-orbit torque (SOT) devices^7–9^. An interfacial PMA stack typically consists of a thin ferromagnetic layer (*e.g*., CoFeB) sandwiched between a heavy metal layer (*e.g*., Ta) and an oxide layer (*e.g*., MgO). The heavy metal layer interface with the ferromagnetic layer is responsible for the spin Hall effect, which is favorable for SOT and skyrmion devices^10,11^. The critical switching current (*J*_c0_) should be minimized to decrease the power consumption of perpendicular STT-MRAM and SOT devices. Reducing *J*_c0_ requires the exploration of new materials with low Gilbert damping constant (*α*) and large spin Hall angle (*θ*_SHE_). Meanwhile, a large effective anisotropy (*K*_eff_) is also favorable to maintain thermal stability^12,13^.

In addition, spintronic devices need to sustain operation reliability for processing temperatures as high as 400 °C for their integration with existing CMOS fabrication technologies, providing the standard back-end-of-line process compatibility^14^. Based on this requirement, the magnetic properties of a PMA material should be thermally stable at annealing temperatures (*T*_ann_) up to 400 °C. Unfortunately, Ta/CoFeB/MgO PMA films commonly used in spintronic devices cannot survive with *T*_ann_ higher than 350 °C, due to Ta diffusion or CoFeB oxidation at the interfaces^15–17^. The diffusion of Ta atoms can act as scattering sites to increase the spin-flip probability^18^ and lead to a higher Gilbert Damping constant (*α*), a measure of the energy dissipation from the magnetic precession into phonons or magnons^19^.

Modifying the composition of thin-film stacks can prevent heavy metal diffusion, which is beneficial to both lowering *α* and improving thermal stability^20^. Along this line, new interfacial PMA stacks have been developed, such as Mo/CoFeB/MgO, to circumvent the limitation on device processing temperatures^21,22^. While Mo/CoFeB/MgO films can indeed exhibit PMA at temperatures higher than 400 °C, they cannot be used for SOT devices due to the weak spin Hall effect of the Mo layer^21,22^. Recently, W/CoFeB/MgO PMA thin films have been proposed because of their PMA property at high post-annealing temperature^23^, and the large (negative) spin Hall angle of the W layer (*θ*_SHE_ ≈ −0.30)^24^, which is twice that of a Ta layer (*θ*_SHE_ ≈ −0.12 ~ −0.15)^9,25^. While there have been a few scattered studies demonstrating the promise of fabricating SOT devices using the W/CoFeB/MgO stacks, special attention has been given to their PMA properties and functionalities as SOT devices^26,27^, or the damping of in-plane W/CoFeB stacks^28^. A systematic investigation is lacking on the effect of *T*_ann_ on *α* of W/CoFeB/MgO PMA thin films with perpendicular anisotropy, as well as the physical mechanisms that alter *α* after post-annealing.

## Methods

### Sample preparation and magnetic characterization

In this work, we grow a series of W(7)/Co_20_Fe_60_B_20_(1.2)/MgO(2)/Ta(3) thin films on Si/SiO_2_(300) substrates (thickness in nanometers) with a magnetron sputtering system (<5 × 10^−8^ Torr). These films are post-annealed at varying temperatures (*T*_ann_ = 250–350 °C for 1 hour, 400 °C for 30 minutes) within a high-vacuum furnace (<1 × 10^−6^ Torr). After post-annealing, the magnetic properties and damping constants of these films are systematically investigated as a function of *T*_ann_. For comparison, a reference sample of Ta(7)/Co_20_Fe_60_B_20_(1.2)/MgO(2)/Ta(3) is also prepared to examine the effect of seeding layer to the damping constant of these PMA films. The effective saturation magnetization (*M*_s_) and anisotropy of these films are measured with the Vibrating Sample Magnetometer (VSM) module of a Physical Property Measurement System (Quantum Design, DynaCool).

### TR-MOKE measurements and data reduction

The magnetization dynamics of these PMA thin films are determined using the all-optical Time-Resolved Magneto-Optical Kerr Effect (TR-MOKE) method^29–34^. This pump-probe method utilizes ultra-short laser pulses to thermally demagnetize the sample and probe the resulting Kerr rotation angle (*θ*_K_). In the polar-MOKE configuration, *θ*_K_ is proportional to the change of the out-of-plane component of magnetization^35^. Details of the TR-MOKE setup are provided in Section S2 of the SI.

The TR-MOKE signal is fitted to the equation

$${\theta }_{{\rm{K}}}=A+B{e}^{-t/C}+D\,\sin (2\pi \,ft+\phi ){e}^{-t/\tau },$$

where *A*, *B*, and *C* are the offset, amplitude, and exponential decaying constant of the thermal background, respectively. *D* denotes the amplitude of oscillations, *f* is the resonance frequency, *φ* is a phase shift (related to the demagnetization process), and *τ* is the relaxation time of magnetization precession. Directly from TR-MOKE measurements, an effective damping constant (*α*_eff_) can be extracted based on the relationship *α*_eff_ = 1/(*2πfτ*). However, *α*_eff_ is not an intrinsic material property; rather, it depends on measurement conditions, such as the applied field direction (*θ*_H_), the magnitude of the applied field (*H*_ext_), and inhomogeneities of the sample (*e.g*. local variation in the magnetic properties of the sample)^36,37^.

To obtain the Gilbert damping constant, the inhomogeneous contribution needs to be removed from *α*_eff_, such that the remaining value of damping is an intrinsic material property and independent of the measurement conditions. To determine the inhomogeneous broadening in the sample, the effective anisotropy field (${H}_{k,\mathrm{eff}}=2{K}_{{\rm{eff}}}/{M}_{{\rm{s}}}$) needs to be pre-determined from either (1) the magnetic hysteresis loops; or (2) the fitting results of *f* vs. *H*_ext_ obtained from TR-MOKE. The resonance frequency, *f*, can be related to *H*_ext_ through the Smit-Suhl approach by identifying the second derivatives of the total magnetic free energy, which combines a Zeeman energy, an anisotropy energy, and a demagnetization energy^38–40^. For a perpendicularly magnetized thin film, *f* is defined by Eqs 1–4^40^.

1 $$f=\frac{\gamma }{2\pi }\sqrt{{H}_{1}{H}_{2}},$$

2 $${H}_{1}={H}_{{\rm{ext}}}\,\cos (\theta -{\theta }_{{\rm{H}}})+{H}_{{\rm{k}},{\rm{eff}}}{\cos }^{2}(\theta ),$$

3 $${H}_{2}={H}_{{\rm{ext}}}\,\cos (\theta -{\theta }_{{\rm{H}}})+{H}_{{\rm{k}},{\rm{eff}}}\,\cos (2\theta ),$$

4 $$2{H}_{{\rm{ext}}}\,\sin ({\theta }_{{\rm{H}}}-\theta )={H}_{{\rm{k}},{\rm{eff}}}\,\sin (2\theta ).$$

This set of equations permits calculation of *f* with the material gyromagnetic ratio (*γ*), *H*_ext_, *θ*_H_, *H*_k,eff_, and the angle between the equilibrium magnetization direction and the surface normal (*θ*, determined by Eq. 4). The measured values of *f* as a function of *H*_ext_ can be fitted to Eq. 1 by treating *γ* and *H*_k,eff_ as fitting parameters. To minimize the fitting errors resulting from the inhomogeneous broadening effect that is pronounced at the low fields, we use measured frequencies at high fields (*H*_ext_ > 10 kOe) to determine *H*_k,eff_.

With a known value of *H*_k,eff_, the Gilbert damping constant of the sample can be determined through a fitting of the inverse relaxation time (1/*τ*) to Eq. 5. The two terms of Eq. 5 take into account, respectively, contributions from the intrinsic Gilbert damping of the materials (first term) and inhomogeneous broadening (second term)^36^:

5 $$\frac{1}{\tau }=\frac{1}{2}\alpha \gamma ({H}_{1}+{H}_{2})+\frac{1}{2}|\frac{d\omega }{d{H}_{{\rm{k}},{\rm{eff}}}}|{\rm{\Delta }}{H}_{{\rm{k}},{\rm{eff}}},$$

where *H*_1_ and *H*_2_ are related to the curvature of the magnetic free energy surface as defined by Eqs 2 and 3^40,41^. The second term on the right side of Eq. 5 captures the inhomogeneous effect by attributing it to a spatial variation in the magnetic properties (Δ*H*_k,eff_), analogous to the linewidth broadening effect in Ferromagnetic Resonance measurements^42^. The magnitude of *d*ω/*dH*_k,eff_ can be calculated once the relationship of *ω* vs. *H*_ext_ is determined with a numerical method. Both *α* and Δ*H*_k,eff_ (the inhomogeneous term related to the amount of spatial variation in *H*_k,eff_) are determined *via* the fitting of the measured 1/*τ* based on Eq. 5. In this way, we can uniquely extract the field-independent *α*, as an intrinsic material property, from the effective damping (*α*_eff_), which is directly obtained from TR-MOKE and dependent on *H*_ext_.

It should be noted here that the inhomogeneous broadening of the magnetization precession is presumably due to the multi-domain structure of the materials, which becomes negligible in the high-field regime (*H*_ext_ $\gg$ *H*_k,eff_) as the magnetization direction of multiple magnetic domains becomes uniform. This is also reflected by the fact that the derivative in the second term of Eq. 5 approaches zero for the high-field regime^43^.

## Results and Discussion

**Figure 1:**
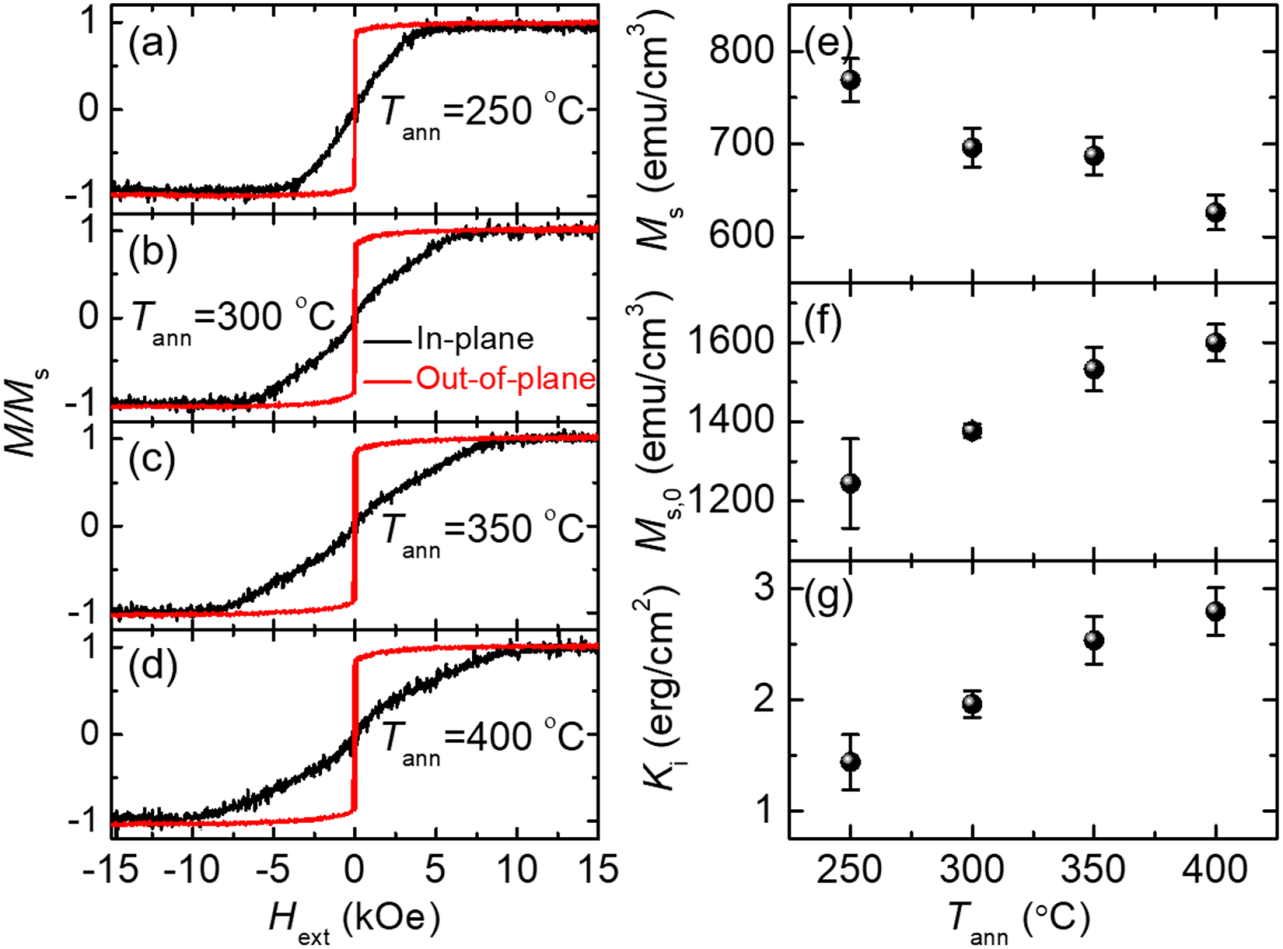
Room temperature magnetic hysteresis loops of W/CoFeB/MgO PMA thin films post-annealed at (**a**) 250 °C, (**b**) 300 °C, (**c**) 350 °C, and (**d**) 400 °C. Black and red curves denote external magnetic field (*H*_ext_) applied along and perpendicular to the film plane, respectively. (**e**–**g**) Plots of the effective saturation magnetization (*M*_s_), the intrinsic saturation magnetization (*i.e*., excluding the effect of the dead layer, *M*_s,0_), and the interfacial anisotropy (*K*_i_) as functions of *T*_ann_.

Figure 1 plots the magnetic hysteresis loops and associated magnetic properties extracted from VSM measurements. With the increase of *T*_ann_, *M*_s_ for the W/CoFeB/MgO films decreases from ~780 to ~630 emu cm^−3^ (Fig. 1e). The PMA in the W/CoFeB/MgO films is dominated by the interface anisotropy (*K*_i_), which increases from 1.4 to 2.8 erg cm^−2^ (excluding the dead-layer thickness effect) with *T*_ann_ up to 400 °C (Fig. 1g). If the film thicknesses are corrected by subtracting the dead layer, *K*_i_ will change from 1.3 to 1.6 erg cm^−2^ as *T*_ann_ increases from 250 to 400 °C, which agrees better with literature values^44^. Details about the determination of *K*_i_ including the dead-layer effect are provided in Section S1 of the Supplementary Information (SI).

**Figure 2:**
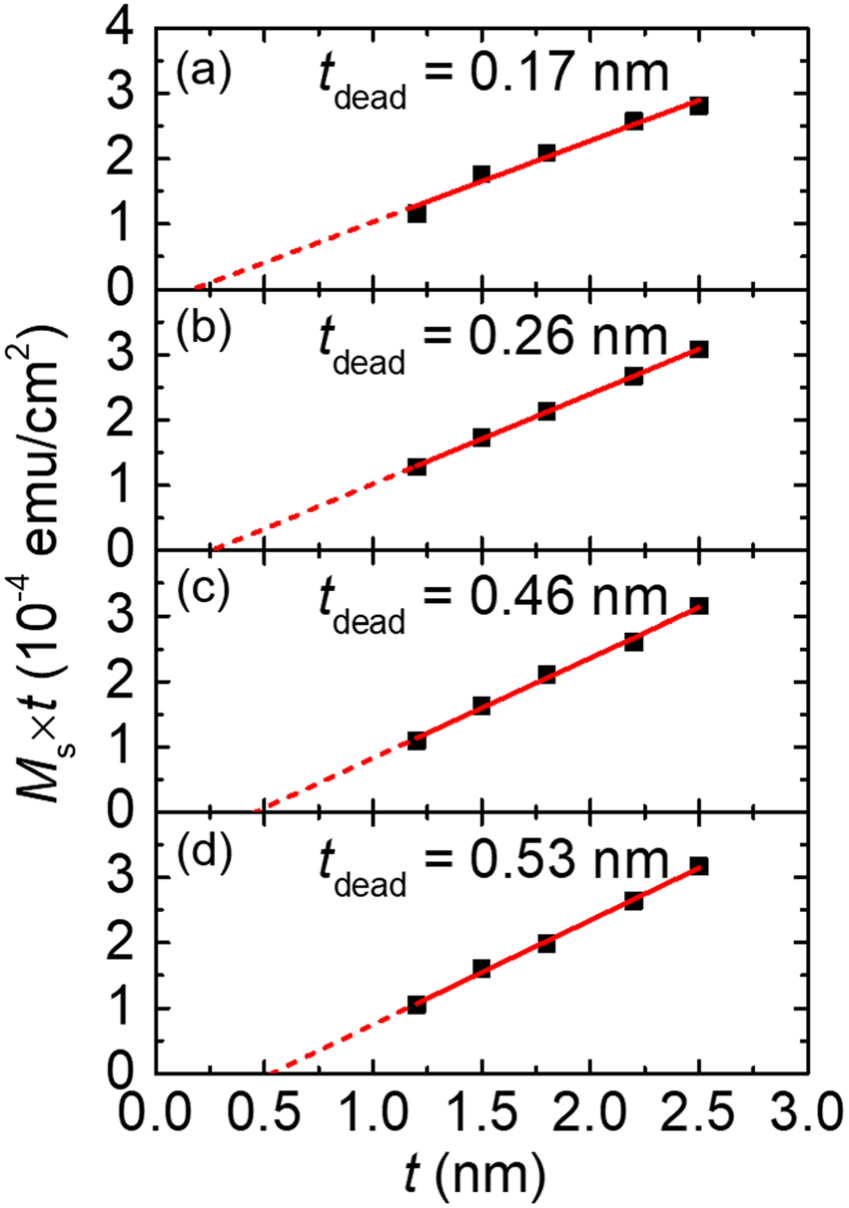
The dead-layer extraction results. (**a**–**d**) represent the series of samples annealed at 250, 300, 350, and 400 ^ο^C respectively. The *t*_dead_ value is the extrapolated *x*-axis intercept from the linear fitting of the thickness-dependent saturation magnetization area product (*M*_s_ × *t*).

We attribute the decrease of *M*_s_ at high *T*_ann_ to the growth of a dead layer at the CoFeB interfaces, which becomes prominent at higher *T*_ann_. To quantitatively determine the thickness of the dead layer as *T*_ann_ increases, we prepare four sets of PMA stacks of W(7)/CoFeB(*t*)/MgO(2)/Ta(3). One set contains five stacks with varying thicknesses for the CoFeB layer (*t* = 1.2, 1.5, 1.8, 2.2, and 2.5 nm) and is post-annealed at a fixed *T*_ann_. Four *T*_ann_ of 250, 300, 350, and 400 °C are used for four sets of the PMA stacks, respectively. The annealing conditions are the same as those for the W(7)/CoFeB(1.2)/MgO(2)/Ta(3) samples discussed previously. We measure the magnetic hysteresis loops of these samples using VSM and plot their saturation magnetization area product (*M*_S_ × *t*) as a function of film thickness (*t*) in Fig. 2. Linear extrapolation of the *M*_S_ × *t* data provides the dead-layer thickness, at which the magnetization reduces to zero as illustrated by the *x*-axis intercept in Fig. 2. The slope of the linear fit also provides intrinsic saturation magnetization (*M*_s,0_), which corresponds to the saturation magnetization after the removal of the dead-layer effect. The values of *M*_s0_ (Fig. 1f) show an increasing trend with *T*_ann_ from ~1300 to ~1600 emu cm^−3^, which agrees well with previous measurement results for W/CoFeB/MgO films^44^.

**Figure 3:**
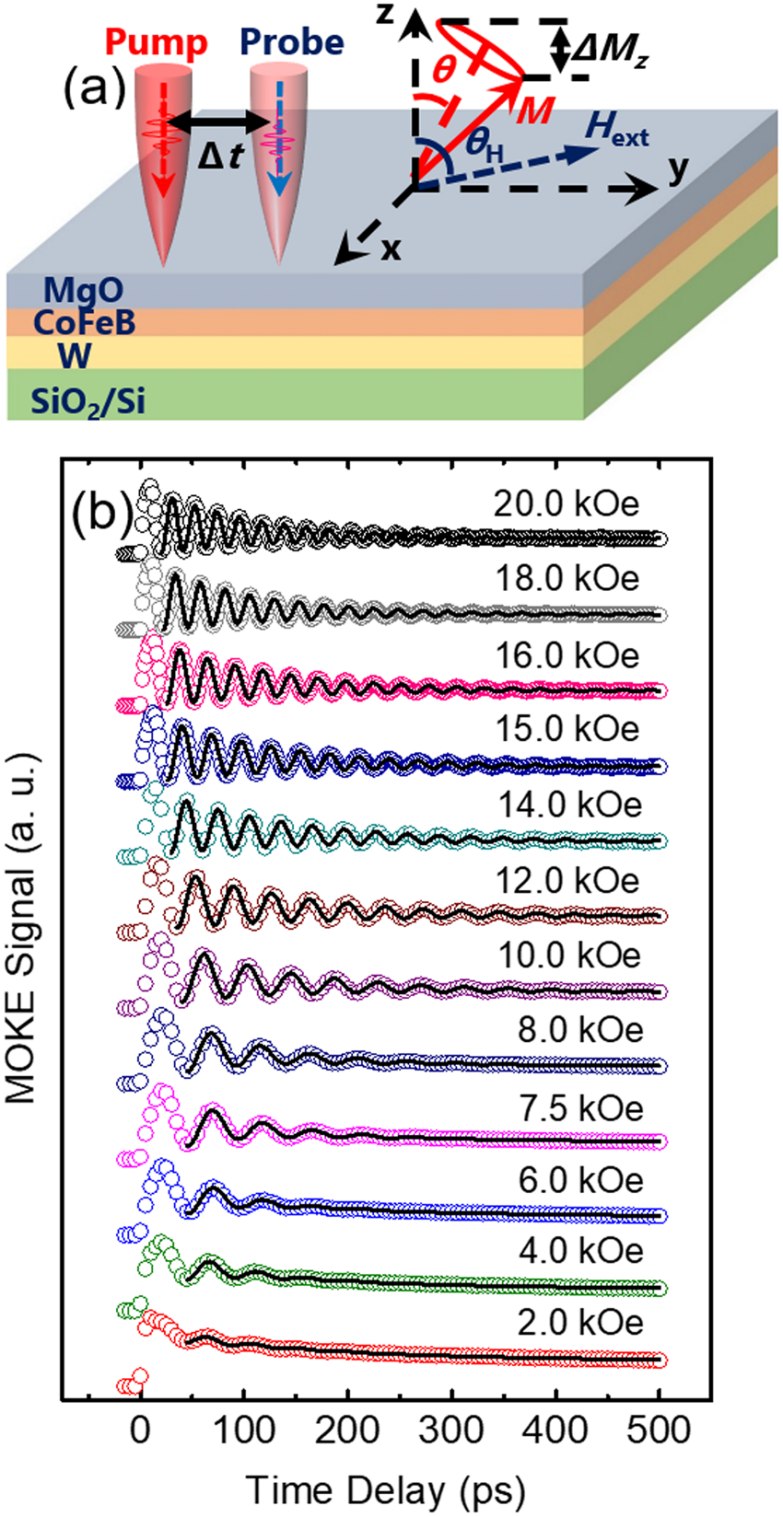
(**a**) Definition of the parameters and angles used in TR-MOKE experiments. The red circle indicates the magnetization precession. *θ* is the equilibrium direction of the magnetization. *θ*_K_ is measured by the probe beam at a given time delay (Δ*t*). (b) The TR-MOKE data (open symbols) and model fitting of *θ*_K_ (black curves) for the 400 °C sample at 76°, for varying *H*_ext_ from 2.0 to 20 kOe.

Figure 3a depicts the schematic of polar TR-MOKE experiments with the definition of several parameters and angles that are important for the data reduction (Section 2.2). Figure 3b plots the TR-MOKE signals (symbols) and the model fitting of *θ*_K_ (black lines) as functions of time delay for the 400 °C W/CoFeB/MgO sample at an external field angle of *θ*_H_ = 76°. To determine the values of *H*_k,eff_ and *α*, the TR-MOKE measurements are conducted at varying *H*_ext_ from 2 to 20 kOe. Both the *f* and *τ* of the measured oscillations, resulting from the magnetization precession, depend greatly on *H*_ext_ as predicted by Eqs 1 and 5.

**Figure 4:**
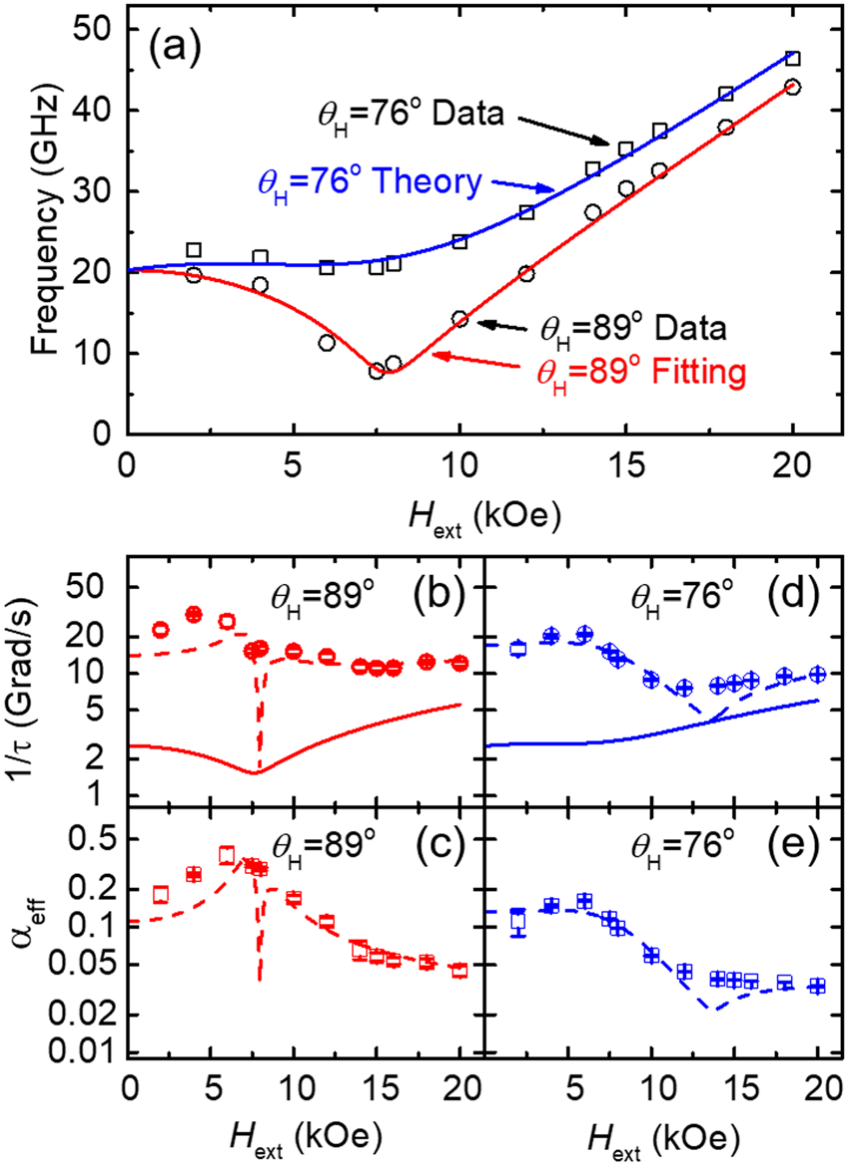
(**a**) Measured *f* vs. *H*_ext_ results for the 400 ^ο^C sample at *θ*_H_ = 89° (open circles) and *θ*_H_ = 76° (open squares) and corresponding modeling at *θ*_H_ = 89° (red line) and *θ*_H_ = 76° (blue line). (**b**) The measured inverse of relaxation time (1/*τ*) at *θ*_H_ = 89° (open symbols) and the fitting of 1/*τ* based on Eq. 5 (dotted line). For reference, the first term of 1/*τ* in Eq. 5 is also plotted (solid line), which accounts for the contribution from the Gilbert damping only. (**c**) *α*_eff_ as a function of *H*_ext_ for *θ*_H_ = 89° (red circles). The dotted line shows the predicted *α*_eff_ using the *α* extracted from the fitting of 1/*τ*. (**d**,**e**) depict similar plots of 1/*τ* and *α*_eff_ for *θ*_H_ = 76°.

By repeating this measurement at varying *θ*_H_, we can show that *α* is an intrinsic material property, independent of *θ*_H_. Figure 4a plots the resonance frequencies derived from TR-MOKE and model fittings for the 400 °C sample at two field directions (*θ*_H_ = 76° and 89°). For the data acquired at *θ*_H_ = 89°, a minimum *f* occurs at *H*_ext_ ≈ *H*_k,eff_. This corresponds to the smallest amplitude of magnetization precession, when the equilibrium direction of the magnetization is aligned with the applied field direction at the magnitude of *H*_k,eff_^40^. The dip at this local minimum diminishes when *θ*_H_ decreases, as reflected by the comparison between the red (*θ*_H_ = 89°) and blue (*θ*_H_ = 76°) lines in Fig. 4a. With the *H*_k,eff_ extracted from the fitting of frequency data with *θ*_H_ = 89°, we generate the plot of theoretically predicted *f* vs. *H*_ext_ (*θ*_H_ = 76° theory, blue line in Fig. 4a), which agrees well with experimental data (open squares in Fig. 4a).

The inverse relaxation time (1/*τ*) should also have a minimum value near *H*_k,eff_ for *θ*_H_ = 89° if the damping was purely from Gilbert damping (as shown by the solid lines in Fig. 4b,d); however, the measured data do not follow this trend. Adding the inhomogeneous term (dotted lines in Fig. 4b,d) more accurately describes the field dependence of the measured 1/*τ* (open symbols in Fig. 4b,d) It should be noted that the dip of the predicted 1/*τ* occurs when the frequency derivative term in Eq. 5 approaches zero; however, this is not captured by the measurement. Figure 4c,e depict the field-dependent effective damping (*α*_eff_) calculated using the Gilbert damping (*α*) extracted from fitting the measured 1/*τ*.

**Figure 5:**
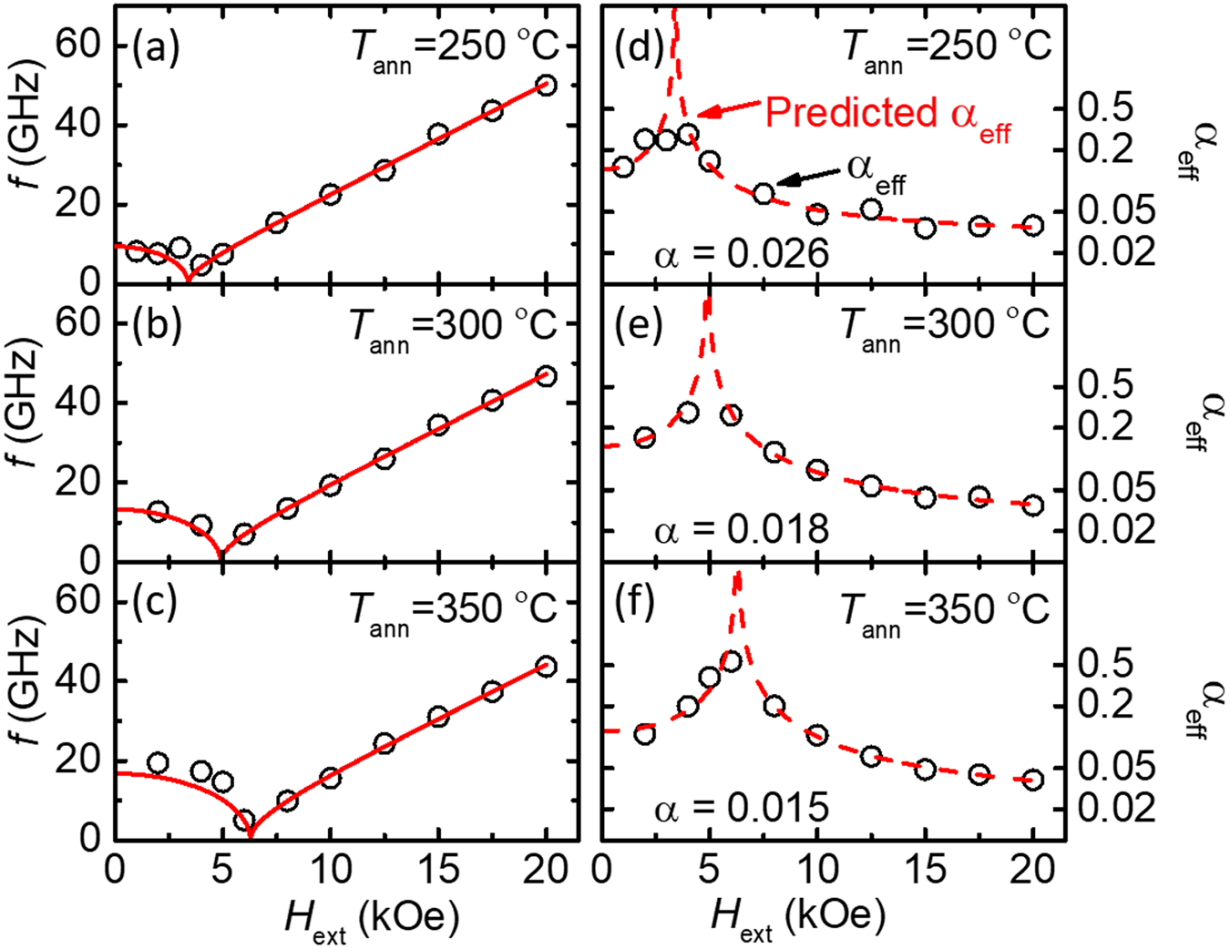
Results for *f* (**a**–**c**) and *α*_eff_ (**d**–**f**), on a log scale, for individual samples (excluding the 400 °C sample, which is discussed in Fig. 4). The fitting for *f* is shown as a solid red line. The dashed line in (**d**–**f**) indicates the predicted *α*_eff_ from the values of *α* extracted from fitting 1/*τ.* All three samples are measured at *θ*_H_ = 90°.

With the knowledge that the value of *α* extracted with this method is the intrinsic material property, we repeat this data reduction technique for the annealed W/CoFeB/MgO samples discussed in Fig. 1. The symbols in Fig. 5 represent the resonance frequencies and damping constants (both effective damping and Gilbert damping) for all samples measured at *θ*_H_ ≈ 90°. The fittings for the resonance frequency based on Eq. 1 (red lines) are also shown to demonstrate the good agreement between our TR-MOKE measurement and theoretical prediction. The uncertainties of *f*, *τ*, and *H*_k,eff_ are calculated from the least-squares fitting uncertainty and the uncertainty of measuring *H*_ext_ with the Hall sensor.

**Figure 6:**
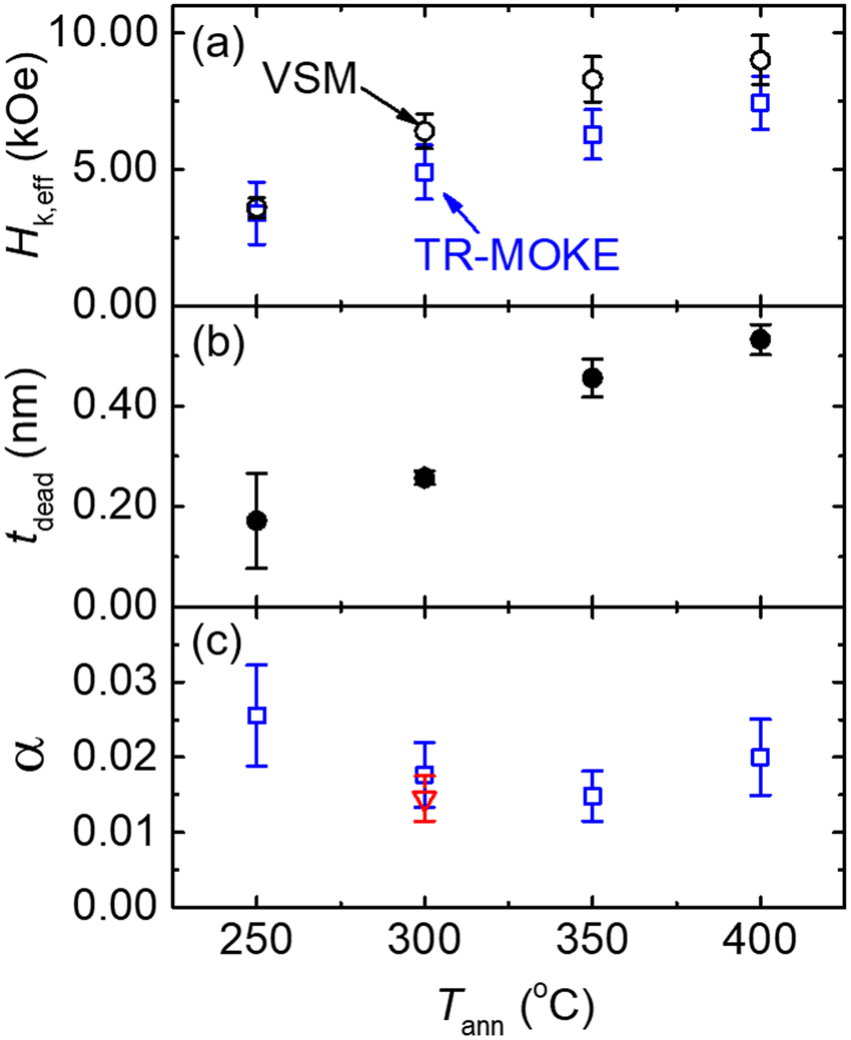
Summary of the magnetic properties of W-seeded CoFeB as a function of *T*_ann_. (**a**) The dependence of *H*_k,eff_ on *T*_ann_ obtained from both the VSM (black open circles) and TR-MOKE fitting (blue open squares). (**b**) The dependence of dead-layer thickness on *T*_ann_. Error bars are from standard error from a linear fit. (**c**) Damping constants as a function of *T*_ann_. The minimum damping constant of *α* = 0.015 occurs at 350 °C. The values for the all samples are obtained from measurements at *θ*_H_ ≈ 90°. For comparison, *α* of the reference Ta/CoFeB/MgO PMA sample annealed at 300 °C is also shown as a red triangle in Fig. 6c.

The summary of the anisotropy and damping measured via TR-MOKE is shown in Fig. 6. Figure 6a plots *H*_k,eff_ obtained from VSM (black open circles) and TR-MOKE (blue open squares), both of which exhibit a monotonic increasing trend as *T*_ann_ becomes higher. Discrepancies in *H*_k,eff_ from these two methods can be attributed to the difference in the size of the probing region, which is highly localized in TR-MOKE but sample-averaged in VSM. Since *H*_k,eff_ determined from TR-MOKE is obtained from fitting the measured frequency for a localized region, we expect these values more consistently describe the magnetization precession than those obtained from VSM. The increase in *H*_k,eff_ with *T*_ann_ has previously be partially attributed to the crystallization of the CoFeB layer^37^. For temperatures higher than 350 °C, this increasing trend of *H*_k,eff_ begins to lessen, presumably due to the diffusion of W atoms into the CoFeB layer, which is more pronounced at higher *T*_ann_. The W diffusion process is also responsible for the decrease in *M*_s_ of the CoFeB layer as *T*_ann_ increases (Fig. 1e). Subsequently, the decrease in *M*_s_ leads to a further-reduced demagnetizing energy and thus a larger *H*_k,eff_.

Similar observation of *M*_s_ has been reported in literature for Ta/CoFeB/MgO PMA structures and attributed to the growth of a dead layer at the heavy metal/CoFeB interface^1^. Figure 6b summarizes *t*_dead_ as a function of *T*_ann_ with *t*_dead_ increasing from 0.17 to 0.53 nm as *T*_ann_ changes from 250 to 400 ^ο^C, as discussed in Section II.

Figure 6c depicts the dependence of *α* on *T*_ann_, which first decreases with *T*_ann_, reaches a minimum of 0.015 at 350 °C, and then increases as *T*_ann_ rises to 400 °C. Similar trends have been observed for Ta/CoFeB/MgO previously (minimum *α* at *T*_ann_ = 300 °C)^37^. We speculate that this dependence of damping on *T*_ann_ is due to two competing effects: (1) the increase in crystallization in the CoFeB layer with *T*_ann_ which reduces the damping, and (2) the growth of a dead layer, which results from the diffusion of W and B atoms and is prominent at higher *T*_ann_.

As the amorphous as-deposited CoFeB film begins to form ordered phases at elevated temperatures, the number of scattering sites in the film tend to decrease^45,46^. The increase in crystallinity of the W/CoFeB/MgO film with *T*_ann_ is demonstrated by the XRD analysis detailed in Section S5 of the SI. At *T*_ann_ = 400 °C, the dead-layer formation leads to a larger damping presumably due to an increase in scattering sites (diffused W atoms) that contribute to spin-flip events, as described by the Elliot-Yafet relaxation mechanisms^18^. Additionally, W atoms can increase the spin-orbit coupling and thus the damping as the inter-diffusion increases with *T*_ann_^47^. The observation that our W-seeded samples still sustain excellent PMA properties at *T*_ann_ = 400 °C confirms their enhanced thermal stability, compared with Ta/CoFeB/MgO stacks which fail at *T*_ann_ = 350 °C or higher.

While the damping constants are comparable for the W/CoFeB/MgO and Ta/CoFeB/MgO films annealed at 300 °C, our work focuses on the enhanced thermal stability of W-seeded CoFeB PMA films that can maintain a relatively low damping constant (0.020 at 400 °C). Such an advantage enables W-seeded CoFeB layers to be viable and promising alternatives to Ta/CoFeB/MgO, which is currently widely used in spintronic devices.

## Conclusion

In summary, we deposit a series of W-seeded CoFeB PMA films with varying annealing temperatures up to 400 °C and conduct ultrafast all-optical TR-MOKE measurements to study their magnetization precession dynamics. The Gilbert damping, as an intrinsic material property, is proven to be independent of measurement conditions, such as the amplitudes and directions of the applied field. The damping constant varies with *T*_ann_, first decreasing and then increasing, leading to a minimum of *α* = 0.015 for the sample annealed at 350 °C. Due to the dead-layer growth, the damping constant slightly increases to *α* = 0.020 at *T*_ann_ = 400 °C, which demonstrates the improved enhanced thermal stability of W/CoFeB/MgO over the Ta/CoFeB/MgO structures. This strongly suggests the great potential of W/CoFeB/MgO PMA systems for future spintronic device integration that requires materials to sustain a processing temperature as high as 400 °C.

## Electronic supplementary material

Supplementary Information
