# Supplementary material for: Low Gilbert Damping Constant in Perpendicularly Magnetized W/CoFeB/MgO Films with High Thermal Stability
Source: Sci Rep. 2018 Sep 6;8:13395. doi: 10.1038/s41598-018-31642-9 (PMC6127240; doi:10.1038/s41598-018-31642-9)
Supplement: Supplementary file 1 — Supplementary Information [file 41598_2018_31642_MOESM1_ESM.pdf]

Supplementary Information for
Low Gilbert Damping Constant in Perpendicularly Magnetized W/CoFeB/MgO Films with High Thermal Stability

Dustin M. Lattery,[1†] Delin Zhang,[2†] Jie Zhu,[1] Xudong Hang,[2] Jian-Ping Wang,[2*] and Xiaojia Wang[1*]

[1]Department of Mechanical Engineering, University of Minnesota, Minneapolis, MN 55455, USA

[2]Department of Electrical and Computer Engineering, University of Minnesota, Minneapolis, MN 55455, USA

[†] These authors contributed equally to this work

[*] Email: wang4940@umn.edu & jpwang@umn.edu

S1. SAMPLE PREPERATION AND MAGNETIC CHARACTERIZATION

The film structures of this work are W(7 nm)/$Co_{20}Fe_{60}B_{20}$(t)/MgO(2 nm)/Ta(3 nm) prepared on Si substrates with a 300-nm thermally oxidized SiO_2 layer. The nominal thickness of the CoFeB layer (t) is varied from 1.0 to 2.5 nm. These CoFeB films are post-annealed at different temperatures (T_{ann} = 250 ~ 400 °C) within a high-vacuum furnace (<1×10^{-6} Torr). The magnetic hysteresis loops are shown in Fig. S1.

The determination of the interfacial anisotropy (K_i) requires the effective anisotropy field ($H_{k,eff}$) and the intrinsic saturation magnetization ($M_{s,0}$) as input parameters, predetermined from the magnetic hysteresis measurements with a VSM. $M_{s,0}$ is obtained by finding the slope of the areal saturation magnetization ($M_s \times t$) as a function of thickness (Fig. 2). The uncertainty of $M_{s,0}$ is treated as the standard error of the slope from fitting. To determine $H_{k,eff}$, we perform a linear

fitting of the normalized magnetization (M/M_s) in the negative H_{ext} range. Only data in the intermediate range of H_{ext} are used to avoid multi-domain effects at low fields and saturation effects at high fields. Extrapolating this linear fit to the point where $M/M_s = -1$ provides $H_{k,eff}$. This process is depicted as the blue lines in Fig. S1.

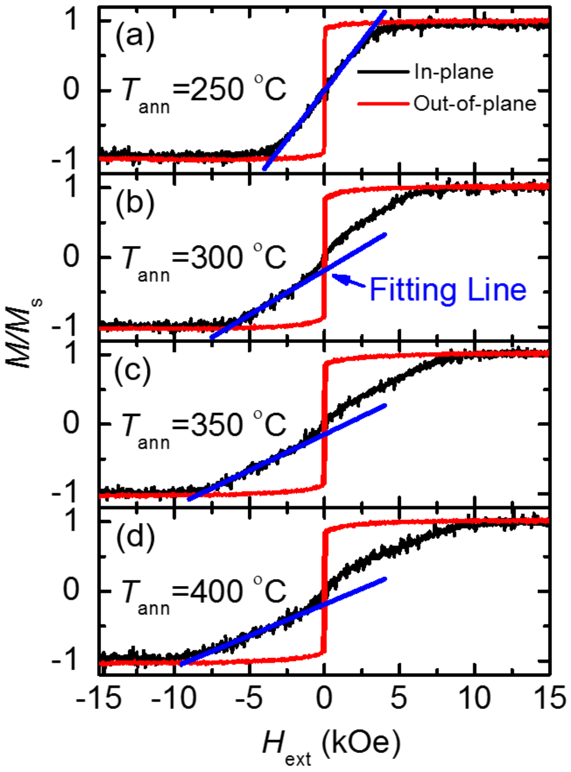

Figure S1. The fitting procedure to extract $H_{k,eff}$ from VSM data. (a-d) represent the series of samples annealed at 250, 300, 350, and 400 °C respectively. Uncertainties in the $H_{k,eff}$ values come from fitting error and measurement uncertainty, and are ~10%.

Once $H_{k,eff}$ and M_s are determined, the effective anisotropy (K_{eff}) can be calculated. This term incorporates the crystalline, interfacial, and shape anisotropy sources as shown in Eqs. S1 and S2[1,2].

$$K_{eff} = \frac{1}{2} H_{k,eff} M_{s,0} \tag{S1}$$

$$K_{\mathrm{eff}} = K_{\mathrm{u}} + \frac{K_{\mathrm{i}}}{t} - 2\pi M_{\mathrm{s},0}^{2} \quad \text{(S2)}$$

Here we adopt the standard that the interfacial anisotropy is predominantly due to the CoFeB/MgO interface, such that the factor of 2 is excluded from the interfacial anisotropy term[3]. For ultrathin CoFeB films (~1 - 2.5 nm in this work), the bulk crystalline anisotropy (K_u) is negligible. This leads to K_{eff} being dominated by K_i, which is effective over the total thickness of the CoFeB layer (t), and the shape (demagnetization) energy (Eq. S2). The values of K_i for all samples range from 1.4 to 2.8 erg cm^{-2}, which is slightly higher than previously reported values for annealed W/CoFeB/MgO films (1.6 to 2.0 erg cm^{-2} for T_{ann} between 300 and 400 °C)[1]. However, these values of K_i are derived based on the total film thickness including the dead layer. Calculation of K_i with the reduced thickness (excluding the dead-layer effect) would result in values of K_i from 1.3 to 1.6 erg cm^{-2} as T_{ann} increases from 250 to 400 °C (from 1.5 to 1.6 erg cm^{-2} for T_{ann} between 300 and 400 °C), which are in better agreement with the results in Ref. 1.

S2. TR-MOKE MEASUREMENT SETUP

The ultrafast time-resolved magneto-optical Kerr effect (TR-MOKE) measurement setup was upgraded from the time-domain thermoreflectance (TDTR) setup described previously[4]. Figure S2a depicts the optical layout of TR-MOKE. A 783-nm Ti:Sapphire laser produces a train of laser pulses of less than 1 ps in duration at a repetition rate of approximately 80 MHz. The laser is separated into a pump beam and probe beam with a polarizing beam splitter. The pump beam is modulated at 9 MHz with an Electro-Optical Modulator, and a delay stage adjusts the length of the pump optical path. Meanwhile, the probe beam is modulated with a mechanical chopper at a frequency of 200 Hz. Both pump and probe beams are co-focused on the sample. The size of the beam spot is controlled with objective lenses of varying magnification. For measurements in this

work, a $1/e^2$ beam spot size with a diameter of 12 μm is used with a 10× objective lens. The pump and probe fluences are set at ~0.3 and 0.1 mJ cm^{-2}, respectively. This relatively low fluence prevents sample damage, and prohibits the decrease of anisotropy and increase in inhomogeneity due to excessive heating[5].

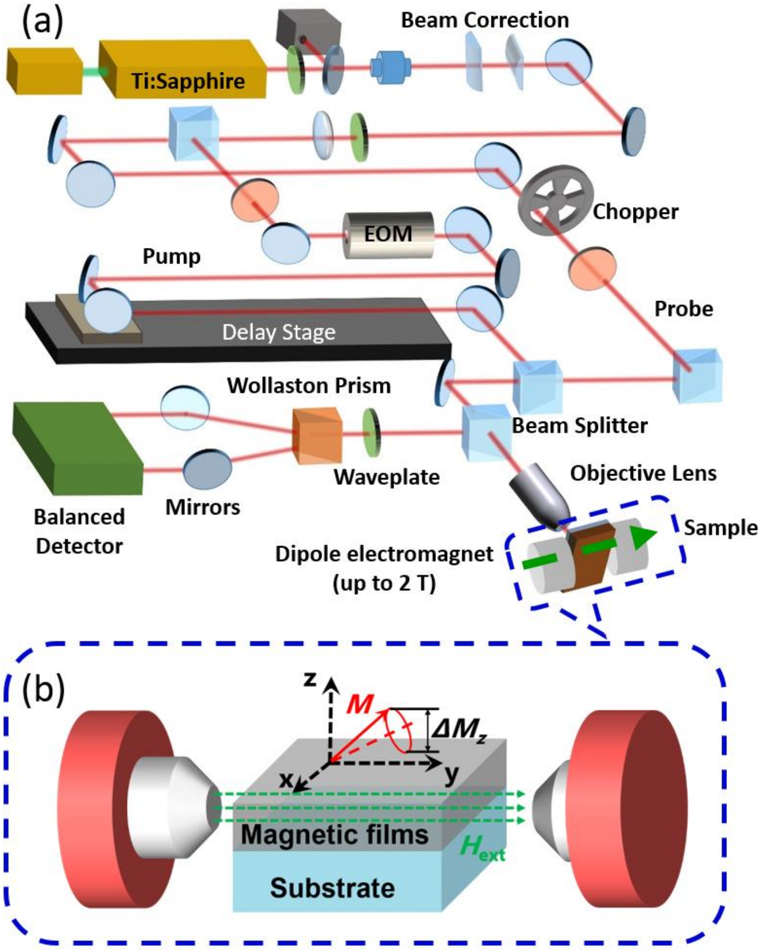

Figure S2. (a) Schematic layout of the TR-MOKE experimental setup depicting the major optical components for measuring the magnetization precession. (b) A magnified view of the sample measurement configuration with respect to the external magnetic field (H_{ext}).

In TR-MOKE measurements, the temperature-dependent variation of the probe polarization (associated with the Kerr rotation angle, θ_K) is monitored, rather than the change of the probe reflectivity as typically used in TDTR. The probe beam travels through a half-wave plate

and is split into two orthogonal linear polarizations with a Wollaston prism. A balanced detector outputs the difference between the intensity of the two polarizations. The time-resolved polar MOKE signal is processed through an RF lock-in amplifier (for the 9 MHz modulation) and a digital lock-in amplifier (for the 200 Hz modulation). For magnetization precession measurements, θ_K is related to the change in the z-component of the magnetization (ΔM_z) as defined in Fig. S2b, which is proportional to the signal captured by the RF lock-in amplifier[6].

An electromagnet produces external magnetic fields up to 2 T. A Hall-probe sensor placed directly behind the sample measures the magnitude of the applied field (H_{ext}). A custom-built rotational stage allows for the control of the applied field direction, as defined by the angle between the sample surface normal and the applied field direction (θ_H). The uncertainty in angular alignment of θ_H is estimated to be approximately ±1°.

S3. ANGULAR DEPENDENCE OF PRECESSIONAL RESONANCE FREQUENCY

To gain some insight into the impact of applied field direction (θ_H) on the resonance frequency (f), we conduct numerical simulations of the field dependent resonance frequency for a representative sample with $\gamma = 1.76\times10^{11}$ rad s^{-1} T^{-1}, $H_{k,eff}$ = 2.5 kOe, and θ_H = 90°, 89°, and 80°. The results of f vs. H_{ext} calculated with Eqs. 1-4 of the main text are presented in Fig. S3. The difference in f between θ_H = 90° and θ_H = 89° is pronounced when the external fields are close to $H_{k,eff}$, but almost negligible elsewhere. For θ_H = 90°, f approaches 0 GHz at $H_{ext} = H_{k,eff}$ as the magnetization theoretically saturates along the direction of H_{ext}. For any other value of θ_H, f experiences a non-zero minimum at $H_{ext} \approx H_{k,eff}$, and this minimum feature of f becomes less apparent when θ_H deviates from 90°. This is demonstrated by the blue curve in Fig. S3 for θ_H = 80°, which shows a very shallow and small dip feature of f when H_{ext} is close to $H_{k,eff}$. For high branch

data at H_{ext} >> $H_{k,eff}$, the slope of f as a function of H_{ext} will always converge to the same value ($\gamma/2\pi$), regardless of θ_H.

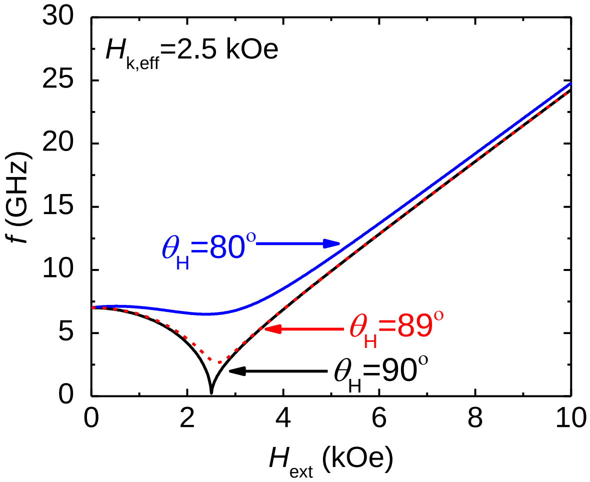

Figure S3. Numerical modeling of f vs. H_{ext} based on Eqs. 1-4 for a representative sample with $\gamma = 1.76\times10^{11}$ rad s^{-1} T^{-1} and $H_{k,eff} = 2.5$ kOe, predicted at θ_H = 90°, 89°, and 80°.

S4. UNCERTAINTY ANALYSIS

The error bars for K_i in Fig. 1g are determined by propagating errors from $H_{k,eff}$, $M_{s,0}$, and the dead layer, all of which have been determined through VSM measurements. We also include an error in the deposited thickness of CoFeB, which is less than 5%. A root sum of squares of errors propagated from various sources (Eqs. S1 and S2) is utilized to determine the overall error of K_i.

Reported uncertainties in f and τ are from the standard error in fitting of the raw TR-MOKE signal (see Fig. 3). The standard error of f is typically negligible and therefore is not shown. The standard errors of individual points of τ (and thus $1/\tau$) depend on the number of oscillations and the signal fluctuation in TR-MOKE measurements, which are typically small as determined from mathematic fitting (Figs. 4b and 4d). The model prediction of $1/\tau$ (and thus α_{eff}) has larger discrepancies with measured values near $H_{k,eff}$ where α_{eff} is large (as shown by the predicted α_{eff}

in Fig. 5), which prompts our primary focus on high-field data ($H_{ext} > H_{k,eff}$) to determine α from the fitting of $1/\tau$.

To estimate the uncertainty of $H_{k,eff}$ from TR-MOKE (blue data points in Fig. 6a), both the standard error from fitting f vs. H_{ext} and the error propagated from the H_{ext} uncertainty are included. The uncertainty of H_{ext} is determined by the instrument resolution of the Hall sensor. Differentiating $H_{k,eff}$ with respect to H_{ext} (Eq. S3), the error propagation from H_{ext} can be approximated. At high fields and $\theta_H = 90°$, a 500 Oe uncertainty of H_{ext} results in <25% uncertainty of $H_{k,eff}$.

$$\frac{\partial H_{k,eff}}{\partial H_{ext}} = 1 + \frac{1}{H_{ext}^2}\left(\frac{2\pi f}{\gamma}\right)^2 \tag{S3}$$

The uncertainty of α also primarily comes from two sources: the standard error from the fitting of $1/\tau$ to measured data ($\Delta\alpha_{SE}$) and the error propagated from $H_{k,eff}$. The best fit for the reported $H_{k,eff}$ for the 250 ºC W/CoFeB/MgO sample is shown as the black line in Fig. S4. The red and blue dashed lines in Fig. S4 portray the fit of $1/\tau$ when $H_{k,eff}$ is adjusted to the upper (blue) and lower (red) limits of its error bar. As mentioned previously, we focus on the high-field data ($H_{ext} \geq 10$ kOe) to prompt the accuracy in determining α. The fittings converge when $H_{ext} \sim 10$ kOe, which indicates that our choice of fitting the data of $H_{ext} \geq 10$ kOe should provide an accurate value of α. The fittings result in α_{high} and α_{low}, which correspond to α determined at the upper and lower limits of $H_{k,eff}$, respectively. The final reported uncertainty in damping ($\Delta\alpha$) is then calculated with Eq. S4, which ranges from 20 to 25%.

$$\Delta\alpha = \sqrt{\left(\frac{\alpha_{high} - \alpha_{low}}{2}\right)^2 + \left(\Delta\alpha_{SE}\right)^2} \tag{S4}$$

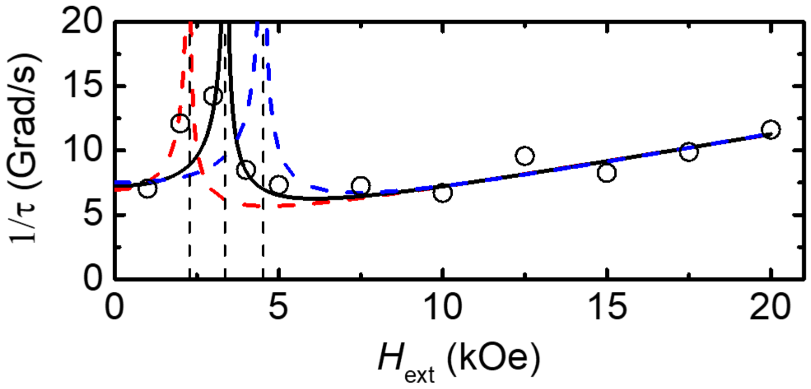

Figure S4. Depiction of the fitting process of $1/\tau$ to determine α and $\Delta\alpha$. The black line indicates the best fit to the measured values of $1/\tau$. The red and blue dotted lines show the fitting of $1/\tau$ when the uncertainty in $H_{k,eff}$ is included.

S5. CHARACTERIZATION OF CRYSTALLINE STRUCTURE AND INTERFACES

X-ray diffraction (XRD) was carried out on a Siemens D5005 diffractometer with Cu-Kα radiation. Since no discernible CoFeB peaks could be identified on the 1.2-nm thick CoFeB films[7], we further fabricated thicker CoFeB films (20 nm in thickness) following a stack structure of Si/SiO_2 sub./MgO(2)/CoFeB(20 nm)/Ta(3) from bottom to top. These thicker films were annealed at 250, 300, 350, and 400 ^{o}C, respectively. The out-of-plane θ-2θ XRD patterns are plotted in Fig. S5.

Due to the challenges in XRD analysis of CoFeB thin films, the CoFeB (110) peak is indiscernible in Fig. S5 for films annealed at low T_{ann}. For films annealed at 400 ^{o}C, the CoFeB (110) peak can be weakly observed, indicating improved crystallinity. In addition, the CoFeB (200) peak can be clearly observed when T_{ann} is 350 ^{o}C or higher, as a direct demonstration of the crystalline CoFeB formation. Both peaks indicate that with the increase in T_{ann}, the CoFeB layer becomes more ordered.

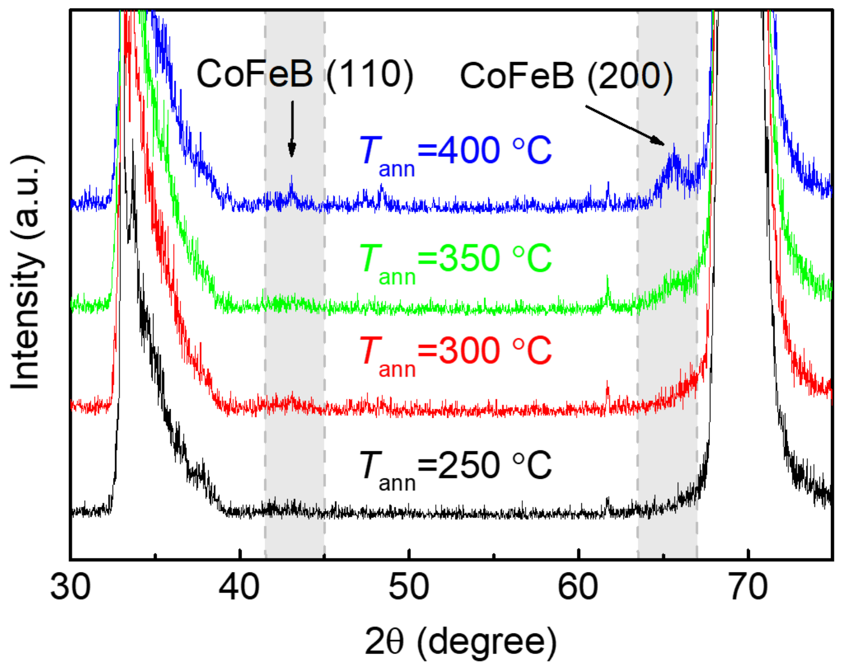

Figure S5. X-ray diffraction θ-2θ scan taken with the scattering vector along the film normal of the CoFeB samples post-annealed at temperatures from 250 to 400 °C.

To determine the interfacial roughness of the W/CFB/MgO thin films, an approximate value of the interfacial roughness was obtained by fitting X-ray reflectivity (XRR) data of the thin films[8]. XRR measurements were carried out on a PANalytical X'Pert high-resolution diffractometer with Cu-Kα radiation. We utilized the GenX software package to fit XRR curves, which provides the information of X-ray scattering length density (SLD)[9]. Despite many possible solutions for the XRR whole curve fitting, these results should reflect the true sample structure, as suggested by the good agreement between the fit and the measured data in Fig. S6. The interfacial roughness values at both interfaces of the CoFeB PMA layer are listed in Fig. S6 (the value in blue is for the CoFeB/MgO interface and the value in red is for the W/CoFeB interface). The XRR data indicate a slight decrease in the roughness of both interfaces with the increase of the post-annealing temperature; however, such reduction in roughness approaches the XRR limit.

Since the decrease in the interfacial roughness is within the XRR limit, we conclude that the interfacial roughness does not make significant contribution to the changes in damping when T_{ann} increases. On the other hand, the W diffusion becomes more active at higher T_{ann}, which tends to increase the damping constant due to its large spin-orbit coupling[10]. The W diffusion dominates when T_{ann} is higher than 350 °C, leading to the increase in damping.

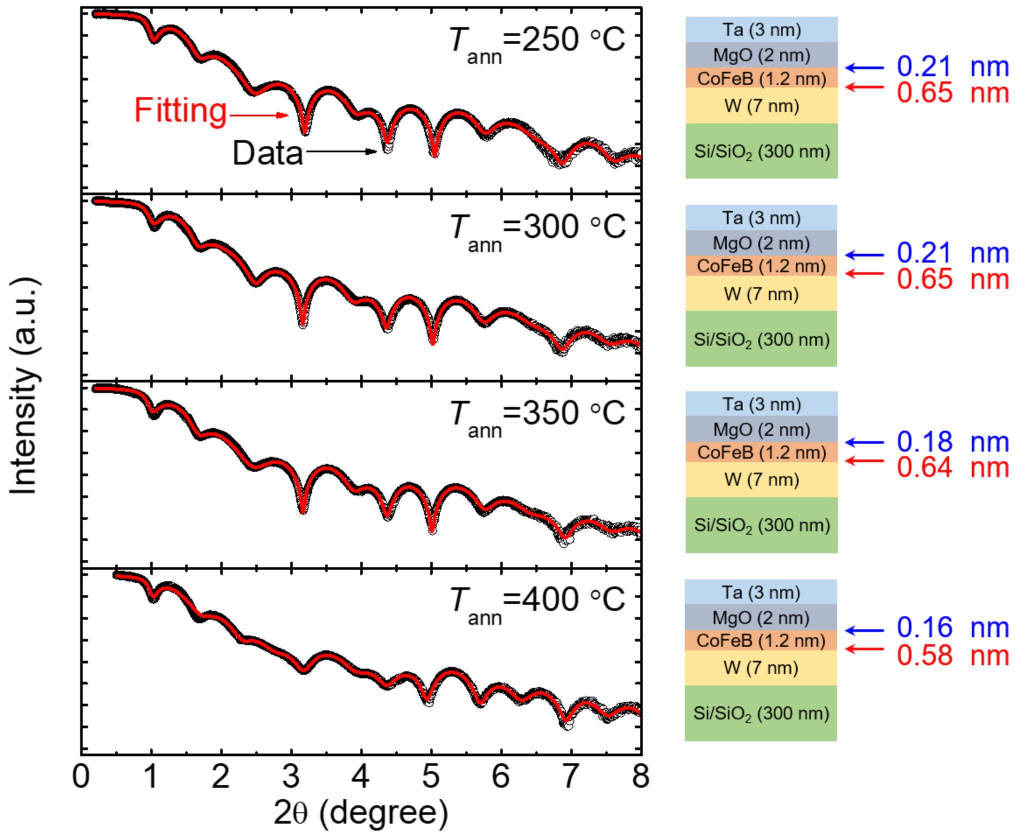

Figure S6. XRR data of the W/CFB/MgO samples post-annealed at T_{ann} = 250, 300, 350, and 400 °C on a log scale. Black circles are the measured data while the red line indicates the GenX fit. Extracted interfacial roughness for the MgO/CoFeB and CoFeB/W interfaces are provided for each T_{ann}.

References

1. An, G.-G. *et al.* Highly stable perpendicular magnetic anisotropies of CoFeB/MgO frames employing W buffer and capping layers. *Acta Mater.* **87**, 259-265 (2015).
2. Almasi, H. *et al.* Perpendicular magnetic tunnel junction with W seed and capping layers. *J. Appl. Phys.* **121**, 153902 (2017).
3. Liu, T., Cai, J. W. & Sun, L. Large enhanced perpendicular magnetic anisotropy in CoFeB/MgO system with the typical Ta buffer replaced by an Hf layer. *AIP Adv.* **2**, 032151 (2012).
4. Kang, K., Koh, Y. K., Chiritescu, C., Zheng, X. & Cahill, D. G. Two-tint pump-probe measurements using a femtosecond laser oscillator and sharp-edged optical filters. *Rev. Sci. Instrum.* **79**, 114901 (2008).
5. Wu, D. *et al.* Laser heating and oxygen partial pressure effects on the dynamic magnetic properties of perpendicular CoFeAlO films. *J. Magn. Magn. Mater.* **409**, 143-147 (2016).
6. Chen, J.-Y. *et al.* Time-resolved magneto-optical Kerr effect of magnetic thin films for ultrafast thermal characterization. *J. Phys. Chem. Lett.* **7**, 2328-2332 (2016).
7. Swamy, G. V., Rout, P. K., Manju, S. & Rakshit, R. K. Resistance minimum and electrical conduction mechanism in polycrystalline CoFeB thin films. *J. Phys. D: Appl. Phys.* **48**, 475002 (2015).
8. Cecot, M. *et al.* Influence of intermixing at the Ta/CoFeB interface on spin Hall angle in Ta/CoFeB/MgO heterostructures. *Sci. Rep.* **7**, 968 (2017).
9. Bjorck, M. & Andersson, G. GenX: an extensible X-ray reflectivity refinement program utilizing differential evolution. *J. Appl. Crystallogr.* **40**, 1174-1178 (2007).
10. He, P. *et al.* Quadratic scaling of intrinsic gilbert damping with spin-orbital coupling in $L1_0$ FePdPt films: experiments and ab initio calculations. *Phys. Rev. Lett.* **110**, 077203 (2013).
